# The Release Rate of Environmental DNA from Juvenile and Adult Fish

**DOI:** 10.1371/journal.pone.0114639

**Published:** 2014-12-05

**Authors:** Atsushi Maruyama, Keisuke Nakamura, Hiroki Yamanaka, Michio Kondoh, Toshifumi Minamoto

**Affiliations:** 1 Faculty of Science and Technology, Ryukoku University, Otsu, Shiga, Japan; 2 Graduate School of Human Development and Environment, Kobe University, Nada-ku, Kobe, Hyogo, Japan; 3 Research Institute for Humanity and Nature, Kita-ku, Kyoto, Japan; Leibniz-Institute of Freshwater Ecology and Inland Fisheries, Germany

## Abstract

The environmental DNA (eDNA) technique is expected to become a powerful, non-invasive tool for estimating the distribution and biomass of organisms. This technique was recently shown to be applicable to aquatic vertebrates by collecting extraorganismal DNA floating in the water or absorbed onto suspended particles. However, basic information on eDNA release rate is lacking, despite it being essential for practical applications. In this series of experiments with bluegill sunfish (*Lepomis macrochirus*), we examined the effect of fish developmental stage on eDNA release rate. eDNA concentration reached equilibrium 3 days after the individual fish were introduced into the separate containers, enabling calculation of the eDNA release rate (copies h^−1^) from individual fish on the assumption that the number of eDNA released from the fish per unit time equals total degradation in the container (copies h^−1^). The eDNA release rate was 3–4 times higher in the adult (body weight: 30–75 g) than in the juvenile group (0.5–2.0 g). Such positive relationship between fish size and eDNA release rate support the possibility of biomass rather than density estimation using eDNA techniques. However, the eDNA release rate per fish body weight (copies h^−1^ g^−1^) was slightly higher in the juvenile than the adult group, which is likely because of the ontogenetic reduction in metabolic activity. Therefore, quantitative eDNA data should be carefully interpreted to avoid overestimating biomass when the population is dominated by juveniles, because the age structure of the focal population is often variable and unseen in the field. eDNA degradation rates (copies l^−1^ h^−1^), calculated by curve fitting of time-dependent changes in eDNA concentrations after fish removal, were 5.1–15.9% per hour (half-life: 6.3 h). This suggests that quantitative eDNA data should be corrected using a degradation curve attained in the target field.

## Introduction

Information on the distribution and biomass of organisms is a crucial component in understanding their ecology and forms the basis for the proper use and management of ecosystems [1]. However, a precise estimation of distribution and biomass is often hindered by complex microhabitat topology and vegetation, particularly in aquatic systems [2]. Although several approaches, such as quadrat and mark-recapture methods, have been established and widely used in the environmental sciences, they are usually destructive to the focal population, labor- and time-consuming, and may not be applicable to some active animals, e.g., fish.

Recently, environmental DNA (eDNA) has been used to document the distributions of aquatic vertebrate species. eDNA is defined as extraorganismal, short, species-specific DNA fragments floating in the water or absorbed onto suspended particles. At present, most eDNA of aquatic vertebrates are considered to exist in or on suspended particles larger than 1.0 µm in diameter, such as cells, cell debris, or mitochondria [3]. Detection of eDNA is expected to increase the accuracy and decrease the cost of surveys [4]. Previous studies have used eDNA to document the presence of amphibians [5]–[7], silver and bighead carp [8], four species of closely-related trout [9], and several freshwater and marine fishes [10]–[12] in a range of water bodies. Presence/absence data can be used to illustrate species distribution and, in the near future, eDNA will be more frequently used to document the presence/absence of aquatic species, particularly in detecting invasive or endangered species.

In addition to the detection of organisms, assuming that aquatic organisms release eDNA into the water either through feces, epidermal mucus, or tissue turnover in proportion with their biomass, the amount of species-specific eDNA in sampled water is expected to indicate the biomass of the focal organism. In fact, several studies suggest that the biomass of focal aquatic organisms can, to some extent, be estimated using eDNA analysis. For example, positive relationships between eDNA concentration and animal density were found in amphibian species [13], [14] and common carp [15].

However, because the eDNA technique for aquatic vertebrate distribution and biomass estimation is still being developed, more basic information is necessary to make this technique applicable to more complex conditions in the field. Importantly, information on the intra-specific variation in the number of eDNA released from the fish per unit time (copies h^−1^; hereafter, eDNA release rate) is lacking, despite it being essential, especially for biomass estimation. Because fish metabolic rates change ontogenetically with growth [16], [17], eDNA release rate is likely to be a function of fish development. Knowledge on the effect of fish developmental stage on eDNA release rate would prevent future researchers from misinterpreting eDNA field data where the age structure of the focal population frequently changes. Such knowledge is necessary not only for the quantitative analysis of populations and communities, but also for the adequate interpretation of presence/absence data because eDNA concentration affects the probability of eDNA detection.

In this study, we conducted a series of aquarium experiments with bluegill sunfish (*Lepomis macrochirus*) to examine the effect of fish developmental stage on eDNA release rate. Our target eDNA was 100 bp fragment of the cytochrome *b* gene, collected by the precipitation method. The eDNA release rate can be calculated on the assumption that it is equal to total degradation (copies h^−1^) when the eDNA concentration (copies l^−1^) has stabilized in the closed system [15], [18]. Hence, the eDNA degradation regression curves were determined using a non-linear model on the time-dependent change in eDNA concentration in the aquariums from which fish had been removed. The eDNA release rate was then compared between juvenile and adult bluegills using aquariums in which a single fish was kept until the eDNA concentration had reached equilibrium.

## Materials and Methods

### Study species and sample fish

The bluegill sunfish (*Lepomis macrochirus*; Rafinesque, 1819) was used as the model fish species in this study because it is one of the most widely distributed freshwater fish species in Japan, introduced from North America in 1960 [19]. The introduction of bluegills has caused ecological problems at both the community and ecosystem level, so the need to monitor the distribution and biomass of this fish is a priority [20].

For the aquarium experiment, bluegills were collected using dip nets and by angling in October, 2012 from a pond (34°58′50″ N, 135°56′40″ E, 5.6 ha) and along the eastern shore in the southern basin of Lake Biwa in Shiga Prefecture, Japan. No endangered or protected species were involved in this study. Fish sampling in the Lake Biwa water system was permitted by the Fisheries Division of Shiga Prefecture, Japan (permission number 24–54). Live fish were brought back to the laboratory and kept in 60 l plastic fish tanks (<21 individuals per tank) at 20°C under a 12-h light: dark cycle and fed chironomid larvae until the experiment started (<60 days). The bluegill aquarium experiments were conducted in accordance with the Invasive Alien Species Act and permitted by the Ministry of Aquaculture, Forestry and Fisheries and the Ministry of the Environment of Japan Government (permission number 09000327).

### Aquarium experiments and water sampling

Ten juveniles and ten adults were selected from those in the fish tanks for the aquarium experiments. Wet weights of the fish were measured prior to the experiment and classified into juvenile and adult groups (0.5–2.0 and 30–75 g in wet body weight, respectively), which correspond to the two major bluegill size classes in the sampling season [19], [21]. The aquarium experiment was conducted using three different-sized fish containers (22×12×13 cm (3.4 l), 40×25×27 cm (27.0 l), and 60×30×36 cm (64.8 l)), of which one size was selected for each individual fish so that it contained aged tap water at approximately 500 times the wet weight of each individual fish (2.0 g fish l^−1^). The constant ratio of water volume to fish weight reduced the inter-group difference in eDNA concentration and hence made the quantitative eDNA collection more accurate. It also reduced the effect of aquarium size on fish behavior. Each group had a negative control (a container with no fish but aged tap water) and 10 replicates. Prior to the experiments, all containers were cleaned using a chlorine bleach solution to remove any bluegill or other organism DNA fragments. These tanks were randomly arranged in the laboratory at 20°C under a 12-h light: dark cycle, which corresponded to field conditions.

The experimental trials were carried out over the period 21^st^ November to 4^th^ December, 2012. One individual was transferred to each container on day 0. The water in the container was continuously aerated and circulated using an air pump and an air stone, but not filtered so as to avoid unintended removal of bluegill DNA fragments. Sample fish were kept in the containers for 7 days and gently removed from the container on day 7. No food was given to the sample fish from 5 days prior to day 0 until the end of each trial to control the effect of feces. Nevertheless, feces were observed in one trial in the adult group; therefore, data from this trial were not included in analysis.

Water samples (15 ml) were collected daily from day 0 to 4 from the surface of water in all containers (10 juveniles and 9 adults) using disposable pipettes. To understand the time-dependent changes in eDNA concentration and confirm that it had stabilized, water samples from five containers (three juveniles and two adults, randomly selected) were analyzed in advance. Based on the results of previous studies [15], [18] and ours, water samples collected on days 3 and 4 (72 and 92 h after fish introduction) from all containers were used as the stabilized eDNA concentration to compare the eDNA release rate between the two groups. Water samples were also collected from the same five containers at 0, 3, 6, 12, 24, 48, 72, and 96 h after the fish were removed on day 7 to determine the degradation curves.

### DNA extraction and real-time quantitative PCR

Immediately after each sampling, 15 ml water samples were mixed with 1.5 ml of 3 M sodium acetate (NaAC; pH 5.2) and 30 ml ethanol and kept in a freezer at −40°C until analysis. The sample was brought to the laboratory of the Research Institute for Humanity and Nature (Kyoto, Japan) for eDNA analysis. The sample solution was centrifuged for 1 h at 10,000×*g* and 4°C, and the supernatants were discarded. The pellets were re-suspended in 200 µl pure water. eDNA was extracted from each 200 µl sample solution using a DNeasy Blood and Tissue Kit (Qiagen, Hilden, Germany). The final DNA solution of 100 µl was analyzed by real-time quantitative polymerase chain reaction (qPCR) as described below. Thus, our samples retained entire DNA fragments in water, including intracellular eDNA encased in cells or cell debris and extracellular eDNA freely floating or absorbed onto suspended particles, whereas some of previous studies collected eDNA with size-selective filters (pore size: 0.45–180 µm) [3], [22]. The condition of the target eDNA may have important implications for the eDNA collection efficiency or degradation curves.

Real-time TaqMan PCR with a StepOne-Plus real-time PCR system (Life Technologies, Carlsbad, CA, USA) was used for eDNA quantification. The mitochondrial cytochrome *b* gene fragments were amplified and quantified with the following primers: bluegill CytB_F (5′- GCCTAGCAACCCAGATTTTAACA-3′), bluegill CytB_R (5′- ACGTCCCGGCAGATGTGT-3′), and bluegill CytB_probe (5′-FAM- CGACATCGCAACTGCCTTCTCTTCAGT-TAMRA-3′) [20]. These primers are specific to bluegills and amplify a 100 bp fragment of the cytochrome *b* gene.

Each TaqMan reaction contained 900 nM of each primer, 125 nM TaqMan probe in 1×PCR master mix (TaqMan Gene Expression Master Mix; Life Technologies), and 2 µl of the DNA solution. The total volume of each reaction mixture was 20 µl. The PCR conditions were as follows: 2 min at 50°C, 10 min at 95°C, and 40 cycles of 15 s at 95°C and 60 s at 60°C. qPCR was performed in triplicate and the mean value was used. PCR products of the target sequences were cloned into a pGEM-T Easy Vector (Promega, Tokyo, Japan) and a dilution series of the plasmid containing 30–30,000 copies was amplified as standard in triplicate in each qPCR plate. The amplification efficiencies and *R* ^2^ values for the standard curves were 91–100% and 0.989–0.997, respectively. Three wells of a no-template negative control were included in each qPCR plate and showed no amplification.

### Determination of eDNA degradation curve

To determine the eDNA degradation regression curves, a non-linear model fitting was conducted on the time-dependent changes in eDNA concentration using data from five containers (three juveniles and two adults) at 0, 3, 6, 12, 24, 48, 72, and 96 h after the fish had been removed. Similarly to previous studies [11], [23], [24], we fit an exponential degradation model, in which the eDNA degradation rate per hour was estimated using an eDNA concentration (*N*; number of copies per liter) and a degradation constant (*ß*), as follows:



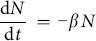
(1)


Solving Equation (1) gives the eDNA concentration at time *t* in hours (*N_t_*) as follows:



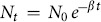
(2)where *N*_0_ is the initial eDNA concentration. The two parameters *N*_0_ and *ß* were determined by the nls function in R ver. 2.12.0 software [25] with an alpha value of 0.05.

The eDNA half-life (*t*_half_), defined as the length of time in which half of the eDNA copies degrade, was calculated by inserting *N_t_* = 1/2 *N*_0_ into Equation (2) as follows:


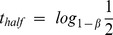
(3)

### Effect of fish developmental stage on eDNA release rate

A differential model assuming that eDNA is released at a constant rate over time was used in a previous study to describe the concentration dynamics of amphibian eDNA in aquariums, because the amphibian eDNA monotonically increased after the introduction of the animals into the aquariums [5], [14]. We did not use such a model because eDNA concentration in fish is known to peak during the acclimation period after introduction to the containers [15]. Instead, in this study, the eDNA release rate was calculated on the assumption that it is in equilibrium to the total eDNA degradation in the container when the eDNA concentration has stabilized.

To confirm stabilization of eDNA concentrations in the containers with fish, a time-dependent change was demonstrated using data from five containers on days 0, 1, 2, 3, and 4 after the bluegills were introduced into the containers. Mean eDNA concentration from days 3 and 4 were used as stabilized values in each container (10 juveniles and 9 adults), based on the time-dependent change in the eDNA concentration in this study and previous studies for similar purposes [15], [18]. The eDNA degradation rate (copies l^−1^ h^−1^) was calculated by Equation (2) with the mean *ß* value obtained from curve fitting and *N* values obtained as the stabilized eDNA concentrations for each trial. The eDNA release rate per individual fish (copies h^−1^) was calculated as the eDNA degradation rate × water volume of each container. The eDNA release rate per fish body wet weight (copies h^−1^ g^−1^) was calculated as per individual fish divided by the mean fish body wet weight measured before and after each trial to the nearest 0.0001 g. The eDNA concentration, eDNA release rate per individual fish, and eDNA release rate per fish body wet weight were all assumed to fit a quasi-Poisson distribution and compared between the juvenile and adult groups by generalized linear models, using the glm function in R ver. 2.12.0 software [25] with alpha values of 0.05.

## Results

### eDNA degradation

**Figure 1:**
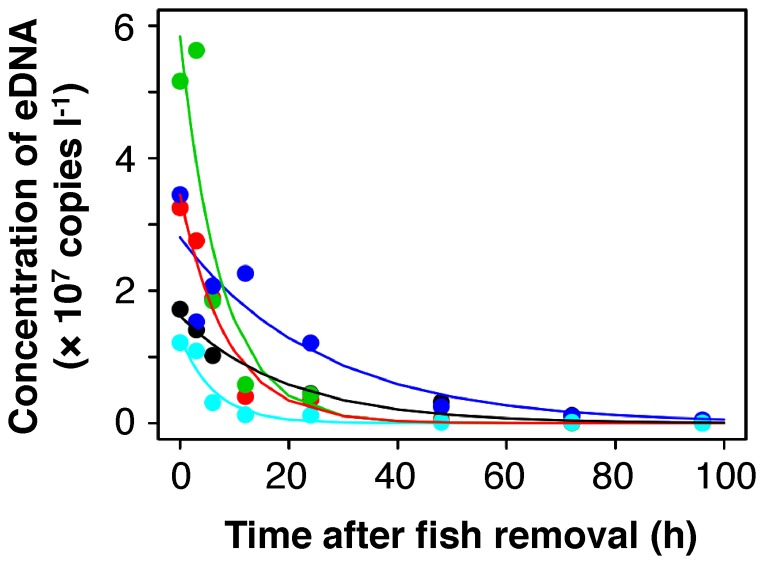
Time-dependent changes in the eDNA concentration after fish removal. Regression curves of the non-linear models are shown. Each color indicates one of five individuals (black and aqua: adult, others: juvenile).

In all five containers, the eDNA concentrations decreased gradually after the fish had been removed (Fig. 1). All non-linear model fittings were statistically significant and the *N*_0_ and *ß* values were calculated as 2.61×10^7^±2.06×10^7^ l^−1^ (mean ±SD, *n* = 5) and 0.104±0.047 h^−1^, respectively (Table 1; Fig. 1). Using the mean *ß* value, the eDNA degradation rate (copies l^−1^ h^−1^) can be estimated by Equation (2) as follows:

**Table 1:** Initial eDNA concentration and degradation constant (*N*_0_ and *ß* respectively; ±SE) estimated by non-linear models fitted to the change in the eDNA concentration after fish removal and fish body wet weight.

*N*_0_ (×10^7^ l^−1^)			*β* (h^−1^)			Weight (g)
3.45±0.29***	0.116±0.020**	0.858
5.84±0.79***	0.132±0.041*	1.074
1.31±0.14***	0.159±0.039**	1.529
1.62±0.11***	0.051±0.010**	30.094
0.82±0.02***	0.060±0.004***	52.466

Significance levels (*t*-test) are indicated by *** (*p*<0.001), ** (*p*<0.01), and * (*p*<0.05).



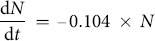
(4)and the eDNA half-life was calculated by Equation (3) to be 6.31 h.

### Effect of fish developmental stage on eDNA release rate

**Figure 2:**
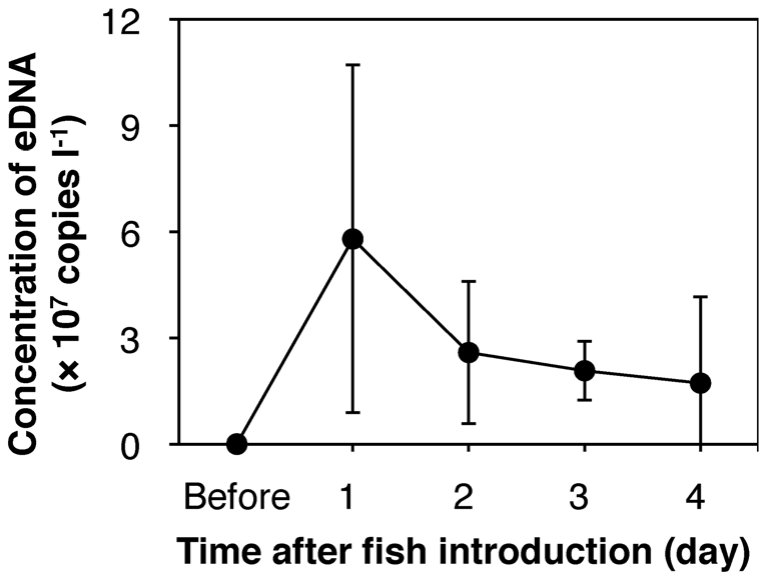
Time-dependent changes in the eDNA concentration after fish introduction. Plots and error bars indicate means and standard deviations of five individuals, respectively.

The eDNA concentration was highest on day 1 (5.80×10^7^±4.92×10^7^ copies l^−1^, mean ± standard deviation) and decreased gradually thereafter, with a small change between days 3 and 4 (Fig. 2).

**Figure 3:**
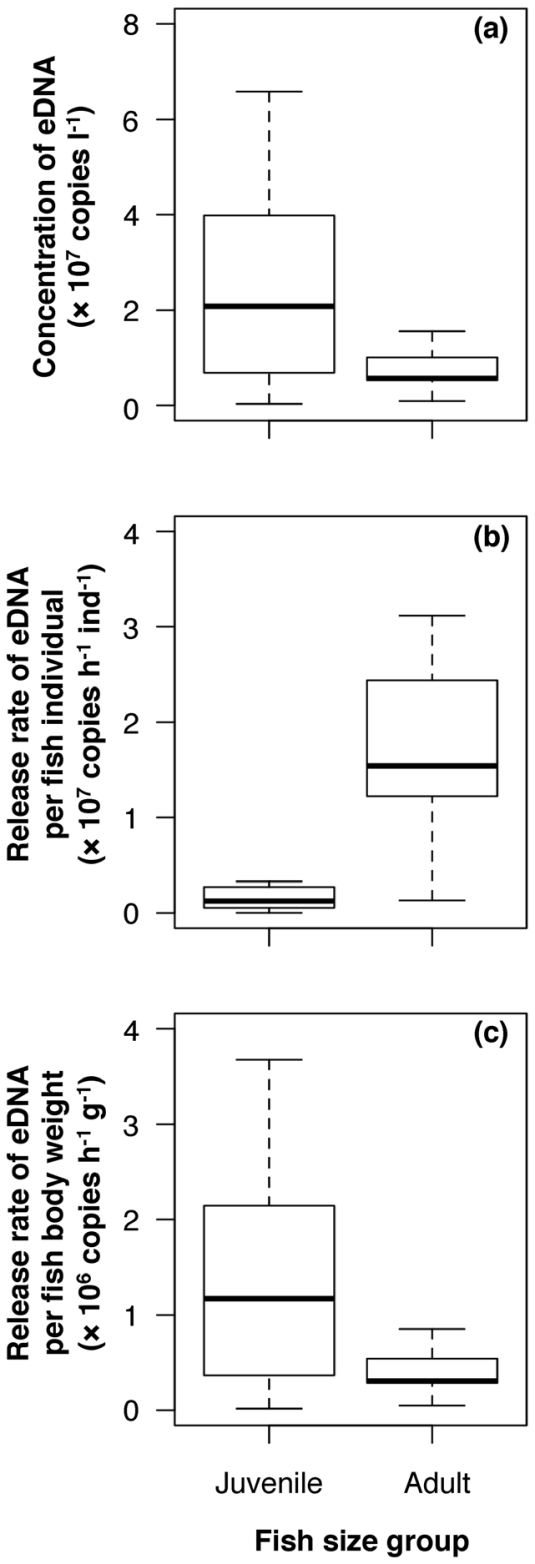
Box plots of the eDNA release compared between juvenile and adult groups. a) Stabilized concentration, b) release rate per individual fish, and c) per fish body weight. Body wet weight was 0.5–2.0 g (*n* = 10) and 30–75 g (*n* = 9), respectively.

The eDNA concentration was approximately 4 times higher in the juvenile group than in the adult group (*t* = −2.739, *p* = 0.0140; Fig. 3a). The eDNA release rate per individual fish was approximately 12 times higher in the adult group than in the juvenile group (*t* = 5.242, *p*<0.0001; Fig. 3b). The eDNA release rate per fish body wet weight was approximately 4 times higher in the juvenile group than in the adult group (*t* = −2.779, *p* = 0.0129; Fig. 3c).

The eDNA copies were detected even from negative controls (containers without fish), suggesting that some amount of rearing water may have contaminated from neighboring containers into control containers. The number of eDNA copies was estimated to be less than 30 copies (per 2 µl PCR sample) in two containers without fish (negative control) on days 3 and 4 and less than 3 copies in all containers prior to fish introduction. These values were less than the quantification limit (30 copies per 2 µl PCR sample  = 1.0×10^5^ copies l^−1^ in the fish container). Thus, it was confirmed that there was no significant contamination between containers.

## Discussion

Although the eDNA technique is expected to become a powerful, non-invasive tool for estimating the distribution and biomass of vertebrates, especially in aquatic ecosystems [4], [6]–[8], [10], [15], [20], the accuracy and limitations of this approach should be considered regarding its practical use in the field. In this study, we examined the effect of fish developmental stage on eDNA release rate, which was calculated based on eDNA degradation regression curves. These are both necessary not only for the accurate estimation of biomass, but also for the adequate interpretation of presence/absence data using the eDNA technique in the field because eDNA concentration affects the probability of eDNA detection.

### Effect of fish developmental stage on eDNA release rate

eDNA concentration peaked on day 1 after introducing the fish into the containers (Fig. 2). This observation indicates that eDNA release rate exceeded degradation rate for a day after fish introduction, probably because of the increased activity of the fish during the acclimation period. Thereafter, eDNA concentration declined by 50–70% and had stabilized by day 3. This initial decline in eDNA concentration was also observed in a previous experiment with common carp [15], the authors concluded that this was because the fish released less eDNA as they became acclimated to the containers and their activity decreased by day 2. However, the relationship between animal activity and eDNA release rate is not yet understood to the best of our knowledge, despite it being essential for understanding of macroorganism eDNA dynamics. Initial eDNA dynamics were obviously different in three amphibian species (Idaho giant salamander, common spadefoot toad and great crested newt), which exhibited monotonic increases after introduction of animals into aquariums [5], [14]. Such differences suggest that special attention should be paid to differences in the pattern of eDNA release between taxonomic groups when statistical models for estimating eDNA dynamics are constructed.

The eDNA release rate per fish body weight was slightly higher in the juvenile group than in the adult group (Fig. 3c), which is likely because of the ontogenetic decrease in the metabolic rate [16], [17]. These differences suggest that the biomass of a population dominated by juveniles may be overestimated when using the eDNA technique, as shown in the comparison of the eDNA concentration (Fig. 3a). Thus, quantitative eDNA data should be carefully interpreted, especially in the field, where age structure or size composition of a fish population can be variable and unseen. At the same time, it is also worth noting that the eDNA release rate per body weight in the juvenile group was <4 times that in the adult group, whereas the body weight of the former was>40 times that of the latter. As a result, the eDNA release rate per individual fish was much higher in the adult group than in the juvenile group because of the larger body size of adult fish. This positive relationship between fish size and eDNA concentration, together with the positive relationship between the number of similar-sized fish and eDNA concentration [15], support the possibility of fish biomass rather than density estimation using the eDNA technique. The estimation error associated with developmental stage may not be totally problematic for practical purposes when compared with the larger estimation errors of traditional methods, which are normally more cost-, labor-, and time-consuming [11]. A rough knowledge of the age structure or size composition of a focal fish population would be useful in preventing researchers from misinterpreting data in the field.

In this study, the eDNA release rates were obtained from the eDNA degradation rates on the assumption that the concentration reached equilibrium by day 3. Apparent change in the mean eDNA concentration after day 2 was approximately 20% per day (Fig. 2), which corresponds to 0.6%–0.8% change per hour. Such small difference is not considered to affect the estimation of the release rates. However, circadian change in the eDNA concentration should be studied in the future studies because it would affect estimations of the release rate.

### eDNA degradation

Information about eDNA degradation is essential to establish proper protocols for eDNA sampling in the field, because the effect of degradation on the quantitative eDNA data needs to be controlled according to the degradation rate [26]. Our non-linear model fitting showed a 5.1–15.9% reduction in eDNA concentration per hour (Table 1, Fig. 1). Degradation was similar to that of a freshwater fish (10.5% for common carp, *Cyprinus carpio*) [23], but more rapid than that of two marine fish (4.6% for European flounder, *Platichthys flesus*; 1.5% for three-spined stickleback, *Gasterosteus aculeatus*) [11]. The large differences in the degradation curves for similar-sized eDNA (100–146 bp) between marine and freshwater fish may have been derived from differences in experimental conditions including temperature (20°C or 15°C) and salinity (freshwater or seawater), which should be considered in future studies focusing on the factors affecting eDNA degradation [5], [23]. The 10% reduction per hour still supports the applicability of the eDNA technique to field surveys. Furthermore, this result suggests that field data for quantitative analysis should be corrected using regression analysis to accurately estimate biomass because the time after sampling until DNA fixation can be as long as an hour and tends to be variable in the field. The differences between studies suggest that a regression curve is necessary using the same primers and same sample water for corrections of eDNA data from the field.

The eDNA half-life was calculated to be 6.3 h, which indicates that more than 90% of eDNA copies degraded within 24 hours. These values suggest the potential of the eDNA approach even for studies on daily migrations of fish. This would be a significant advantage of eDNA techniques, especially for studies of fish, whose high swimming ability has often prevented researchers from collecting accurate data using traditional methods. However, such fine temporal resolution of the eDNA approach has been reported only from experimental conditions[11], [23], which differ from natural settings in many environmental factors that may affect eDNA degradation [5], [23]. Studying the factors affecting eDNA degradation would have great implications for the further application of this technique.

## Conclusions

The positive relationship between fish size and eDNA release rate in this study, together with the positive relationship between the number of similar-sized fish and eDNA concentration [15], support the possibility of biomass estimation using the eDNA technique. However, since the eDNA release rate per fish body weight was variable between juvenile and adult fish, careful attention should be paid to variations in the age structure of the focal fish population when quantitative eDNA data from the field are translated into fish biomass. The eDNA degradation rate was shown to be 10% per hour, which suggests that quantitative eDNA data from the field should be corrected using regression curves to control the effect of degradation after sampling.
