# Correction: The Release Rate of Environmental DNA from Juvenile and Adult Fish

**DOI:** 10.1371/journal.pone.0118727

**Published:** 2015-03-05

**Authors:** The PLOS ONE Staff

There is an error in the third equation. Please view the complete, correct equation here:

$$t_{\text{half}}\;=\;\frac{{log}_{e}2}{\beta }$$

In the penultimate sentence of the Abstract, the last sentence of the first paragraph in the Results section, and the first sentence of the final paragraph of the Discussion section the half-life value is incorrectly calculated as 6.3. The correct half-life value should read 6.7.
