# Correction: The Release Rate of Environmental DNA from Juvenile and Adult Fish

**DOI:** 10.1371/journal.pone.0212145

**Published:** 2019-02-05

**Authors:** Atsushi Maruyama, Keisuke Nakamura, Hiroki Yamanaka, Michio Kondoh, Toshifumi Minamoto

There is an error in Table 1. The correct *N*_0_ and *β* values for the fifth individual (corresponding to the blue individual in Fig 1) are 2.81 ± 0.37*** and 0.039 ± 0.015*, respectively. Please see the corrected Table 1 here.

**Table 1:** Initial eDNA concentration and degradation constant (*N*_0_ and *β* respectively; ± SE) estimated by non-linear models fitted to the change in the eDNA concentration after fish removal and fish body wet weight.

*N*_0_ (× 10^7^ l^–1^)	*β* (h^–1^)	Weight (g)
3.45 ± 0.29***	0.116 ± 0.020**	0.858
5.84 ± 0.79***	0.132 ± 0.041*	1.074
1.31 ± 0.14***	0.159 ± 0.039**	1.529
1.62 ± 0.11***	0.051 ± 0.010**	30.094
2.81 ± 0.37***	0.039 ± 0.015*	52.466

Significance levels (t-test) are indicated by *** (p<0.001),

** (p<0.01) and

* (p<0.05)

As a result of this error, the following sentences should be corrected in the article:

- There is an error in the penultimate sentence of the Abstract section. The correct sentence is: eDNA degradation rates (copies l^–1^ h^–1^), calculated by curve fitting of time-dependent changes in eDNA concentrations after fish removal, were 3.9–15.9% per hour (half-life: 7.0 h).
- In the Results, there are errors in the second and third sentences of the “eDNA degradation” subsection. The correct sentences are: All non-linear model fittings were statistically significant and the *N*_0_ and *β* values were calculated as 3.01 × 10^7^ ± 1.81 × 10^7^ l^–1^ (mean ± SD, *n* = 5) and 0.099 ± 0.052 h^–1^, respectively (Table 1 and Fig 1). Using the mean *β* value, the eDNA degradation rate (copies l^–1^ h^–1^) can be estimated by Equation (2) as follows:

$$\frac{\text{d}N}{\text{d}t}=-0.099\times N$$

and the eDNA half-life was calculated by Equation (3) to be 7.0 h.

- In the Discussion, there is an error in the second sentence of the first paragraph of the “eDNA degradation” subsection. The correct sentence is: Our non-linear model fitting showed a 3.9–15.9% reduction in eDNA concentration per hour (Table 1 and Fig 1).
- In the Discussion, there is an error in the first sentence of the second paragraph of the “eDNA degradation” subsection. The correct sentence is: The eDNA half-life was calculated to be 7.0 h, which indicates that more than 90% of eDNA copies degraded within 24 hours.

In addition, as a result of the errors in Table 1, there are errors in Fig 3. Please see the corrected Fig 3 here.

**Fig 3:**
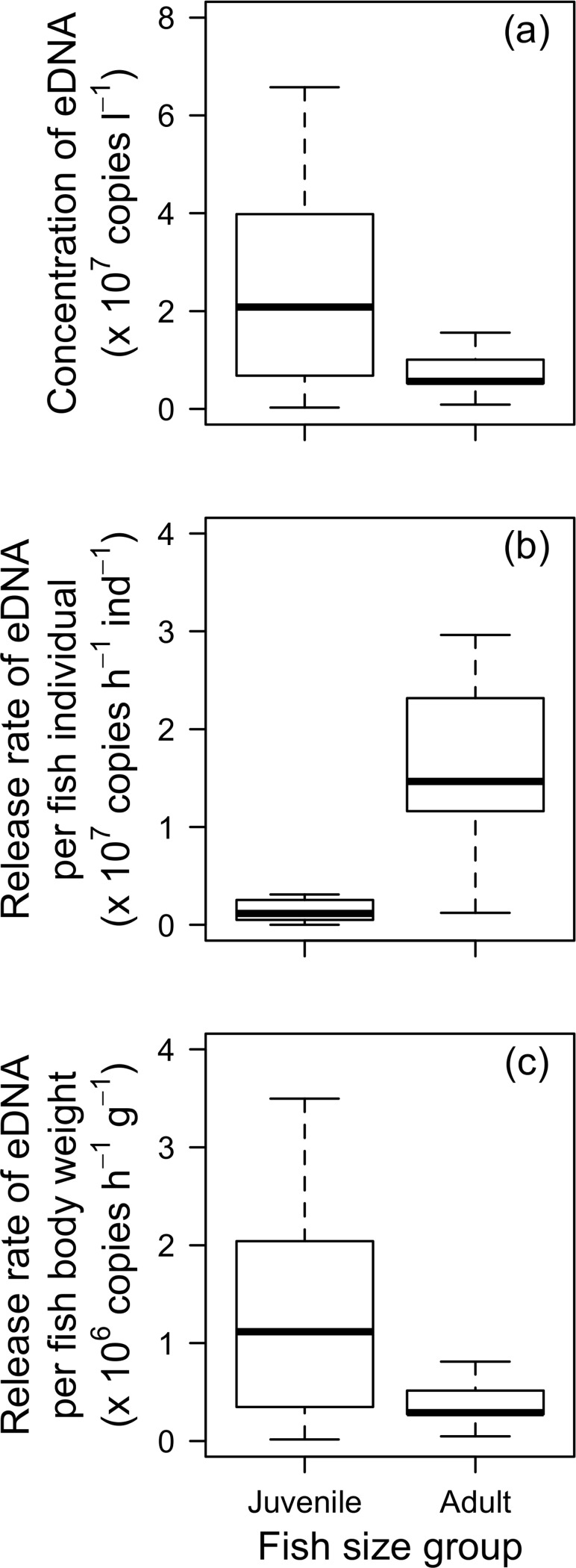
Box plots of the eDNA release compared between juvenile and adult groups. a) Stabilized concentration, b) release rate per individual fish, and c) per fish body weight. Body wet weight was 0.5–2.0 g (*n*  =  10) and 30–75 g (*n*  =  9), respectively.
